# Chemotherapy-initiated cysteine-rich protein 61 decreases acute B-lymphoblastic leukemia chemosensitivity

**DOI:** 10.1007/s00432-024-05692-8

**Published:** 2024-03-26

**Authors:** Pengchong Shi, Zhen Lin, Yanfang Song, Zhaozhong Li, Menglu Zeng, Li Luo, Yingping Cao, Xianjin Zhu

**Affiliations:** 1 Department of Clinical Laboratory, Fujian Medical University Union Hospital, 29 Xinquan Road, Fuzhou, 350001 Fujian China; 2 Department of Clinical Laboratory, Affiliated People Hospital of Fujian University of Traditional Chinese Medicine, 602 Bayiqi Road, Fuzhou, 350001 Fujian China; 3 Department of Laboratory Medicine, Fujian Medical University Union Hospital, 29 Xinquan Road, Fuzhou, 350001 China

**Keywords:** Cyr61, Chemosensitivity, DNA damage, Apoptosis

## Abstract

**Purpose:**

Chemoresistance is a major challenge for acute lymphoblastic leukemia (ALL) treatment. Cysteine-rich protein 61 (Cyr61) plays an important role in drug resistance modulation of tumor cells, and Cyr61 levels are increased in the bone marrow of patients with ALL and contribute to ALL cell survival. However, the effect of Cyr61 on B cell acute lymphoblastic leukemia (B-ALL) cell chemosensitivity and the regulatory mechanisms underlying Cyr61 production in bone marrow remain unknown.

**Methods:**

Nalm-6 and Reh human B-ALL cell lines were used in this study. Cyr61 levels were assessed using quantitative real-time PCR (qRT-PCR), western blot analysis, and enzyme-linked immunosorbent assay. The effect of Cyr61 on B-ALL cell chemosensitivity to daunorubicin (DNR) was evaluated using cell viability and flow cytometry analyses. The regulatory mechanisms of Cyr61 production in bone marrow were examined using qRT-PCR and western blot analysis.

**Results:**

Cyr61 knockdown and overexpression increased and decreased the chemosensitivity of B-ALL cells to DNR, respectively. Cyr61 attenuated chemotherapeutic drug-induced apoptosis by upregulating B cell lymphoma-2. Notably, DNR induced DNA damage response and increased Cyr61 secretion in B-ALL cells through the ataxia telangiectasia mutated (ATM)-dependent nuclear factor kappa B pathway.

**Conclusion:**

DNR induces Cyr61 production in B-ALL cells, and increased Cyr61 levels reduce the chemosensitivity of B-ALL cells. Consequently, targeting Cyr61 or related ATM signaling pathway may present a promising treatment strategy to enhance the chemosensitivity of patients with B-ALL.

## Background

B-acute lymphoblastic leukemia (B-ALL), the most common malignancy in children, is an affliction of uncontrolled B lymphocyte proliferation in the bone marrow (Terwilliger and Abdul-Hay 2017). Although combination chemotherapy has achieved relatively good therapeutic effects in patients with B-ALL, a subset of patients remain who suffer from relapses and chemotherapy resistance (Jabbour et al. 2015). As such, studying the mechanism of drug resistance in B-ALL cells is necessary.

The bone marrow microenvironment plays a pivotal role in leukemia (Hellinger et al. 2019). Abnormal cytokine, chemokine, and matrix protein expression in the bone marrow microenvironment promotes acute leukemia cell survival and migration and reduces sensitivity to chemotherapeutic drugs (Hesler et al. 2016; Cao et al. 2019; Zhu et al. 2016; Acharyya et al. 2012; Huber et al. 2015).

Cysteine-rich protein 61 (Cyr61/CCN1), a member of the CCN (Cyr61/CTGF/NOV) family, plays a pivotal role in maintaining normal bodily physiological functions (Song et al. 2019; Cao et al. 2019; Lum et al. 1993; Hazlehurst et al. 2003; Terada et al. 2012). Cyr61 expression becomes abnormal in tumors, and Cyr61 levels are correlated with poor prognosis in tumors (Sabile et al. 2012; Kok et al. 2010; Jiang et al. 2004; Jeong et al. 2014; D’Antonio et al. 2010; Holcik and Korneluk 2001). Notably, Cyr61 induces drug resistance in tumor cells, such as those in breast cancer and pancreatic adenocarcinoma (Hellinger et al. 2019; Hesler et al. 2016). Cyr61 levels are increased in the bone marrow of patients with ALL, and increased Cyr61 contributes to ALL cell survival and decreases their chemosensitivity to cytarabine (Cao et al. 2019; Zhu et al. 2016). Daunorubicin (DNR), an anthracycline, is widely used as a first-line chemotherapy in ALL. However, the effect of Cyr61 on DNR chemosensitivity has not yet been explored, and the regulatory mechanisms ofCyr61 production in the bone marrow are poorly defined.

Chemotherapy-produced cytokines, growth factors, and matrix proteins may protect tumor cells from therapeutic killing (Liu et al. 2020; Gilbert and Hemann 2010; Tuncali et al. 2018). Doxorubicin induces interleukin (IL-6) production in thymic epithelial cells (Gilbert and Hemann 2010). Furthermore, rituximab induces IL-6 release from diffuse large B cell lymphoma cells, which protect diffuse large B cell lymphoma cells from chemotherapy-induced apoptosis (Zhong et al. 2018). Acharyya et al. reported that the combination of doxorubicin and cyclophosphamide chemotherapy can induce CXCL1/2 expression in breast cancer cells, leading to chemoresistance (Acharyya et al. 2012). In addition, Huber et al. found that docetaxel induces glial cell line-derived neurotrophic factor (GDNF) secretion in human prostate fibroblasts, which promotes resistance to prostate cancer treatment (Huber et al. 2015). However, whether chemotherapy induces Cyr61 release in B-ALL cells remains largely unknown.

In this study, we explored the effect of Cyr61 on DNR chemosensitivity and investigated the effect of DNR on Cyr61 production in B-ALL cells. Collectively, this study is the first to reveal that DNR induces Cyr61 production in B-ALL cells, which in turn promotes B-ALL cell resistance to treatment. Our findings suggest that targeting Cyr61 or related signaling pathways may present a promising option to enhance the chemosensitivity of patients with B-ALL.

## Materials and methods

### Patients and cell lines

Primary leukemic cells were isolated from two patients with B-ALL using a Ficoll gradient as previously described (Zhu et al. 2016). The Nalm-6 and Reh human acute B-lymphocytic leukemia cell lines were cultured with Roswell Park Memorial Institute (RPMI) 1640 medium (HyClone, Logan, UT, USA) supplemented with 10% fetal bovine serum (FBS, Gibco, Carlsbad, CA, USA) and 1% penicillin/streptomycin (HyClone, Logan, UT, USA) at 37 °C and 5% CO_2_. Reh and Nalm-6 both belong to B cell precursor leukemia cell line. Nalm-6 cell line was established from the peripheral blood of a 19-year-old man with acute lymphoblastic leukemia (ALL) in relapse in 1976. Reh was established from the peripheral blood of a 15-year-old girl with acute lymphoblastic leukemia (ALL at first relapse) in 1973; carries t (12;21) leading to ETV6-RUNX1 (TEL-AML1) fusion gene. The cell lines were routinely evaluated for contamination using a mycoplasma contamination test and short tandem repeat (STR) DNA profiling.

### Drugs

DNR (Selleckchem, Houston, TX, USA) was dissolved in dimethyl sulfoxide (DMSO) as per the manufacturer’s instructions. KU55933 (Selleckchem, Houston, TX, USA) was used as ATM phosphorylation inhibitor and PDTC (Selleckchem, Houston, TX, USA) was used as NF‐κB phosphorylation inhibitor. Two phosphorylation inhibitors were dissolved in DMSO as per the manufacturer’s instructions. All the drugs were stored in aliquots at − 20 °C.

### Enzyme‐linked immunosorbent assay (ELISA)

The Cyr61 concentrations in the culture supernatants were determined using human Cyr61 ELISA Systems (R&D Systems, Minneapolis, MN, USA), according to the manufacturer’s instructions. All samples were tested in triplicate, along with three internal quality control plasma and serum samples to assess inter-assay precision.

### Cell viability assay

Cells were cultured in RPMI 1640 medium containing different concentrations of DNR for 24 h. The cell viability was measured using Cell Counting Kit-8 (CCK8, Beyotime Biotechnology, Jiangsu, China), according to manufacturer’s instructions. Cells (5.0 × 10^3^) were seeded into a 96-well plate and cultured at 37 °C for 24 h. CCK8 reagent (10 μL) was added into each well and incubated for an additional 2 h. The optical density (OD) of plates was determined at 450 nm using a microplate reader (BIO-TEK), and the IC 50 (the concentration of a drug that is required for 50% inhibition in vitro) was used to quantitatively indicate the different cell killing capabilities of DNR. Each sample was assayed in triplicate and the experiments were repeated thrice.

### Western blot analysis

Nalm-6 and Reh cells were harvested, washed with ice-cold phosphate-buffered saline (PBS), and added to radioimmunoprecipitation assay (RIPA) lysis buffer for 20 min. Western blotting (protein immunoblotting) was performed as previously described (Zhu et al. 2016; Song et al. 2019; Cao et al. 2019). The following antibodies were used: anti-human cyr61 monoclonal antibody (093G9) was kindly provided by Dr. Ningli Li (Shanghai Jiao Tong University School of Medicine, Shanghai, China) (Lin et al. 2012a, b; Zhang et al. 2009; Zhong et al. 2017), anti‐NF‐κBp65 (4764; Cell Signaling Technology, Danvers, MA, USA), anti-P-NF‐κBp65 (3033; Cell Signaling Technology, Danvers, MA, USA), anti-Bcl-2 (4233; Cell Signaling Technology, Danvers, MA, USA), anti-PI3K/AKT (9272; Cell Signaling Technology, Danvers, MA, USA), anti-p- PI3K/AKT (9271; Cell Signaling Technology, Danvers, MA, USA), anti-p38 MAPK (9212; Cell Signaling Technology, Danvers, MA, USA), anti-p-p38 MAPK (9211; Cell Signaling Technology, Danvers, MA, USA), anti-ERK (p44/42) (9202; Cell Signaling Technology, Danvers, MA, USA), anti-p-ERK (p44/42) (9201; Cell Signaling Technology, Danvers, MA, USA), anti-p-H2A.X (2577; Cell Signaling Technology, Danvers, MA, USA), anti-ATM (2873; Cell Signaling Technology, Danvers, MA, USA), and anti-p-ATM (p44/42) (5883; Cell Signaling Technology, Danvers, MA, USA). AS p-ATM and ATM are very big. To detect them in western blotting is not that easy. It is convention to introduce the molecular weight marker (MM) above and below the detected protein (exception of for ATM because too high for a conventional MM. Therefore, the MM below is enough).

### Quantitative real-time PCR (qRT-PCR)

Total RNA of specimens was extracted using RNAeasy™ Animal RNA Isolation Kit with a spin column (Beyotime, Shanghai, China) according to the manufacturer’s instructions. Total RNA (1 μg) was reverse‐transcribed into first‐strand cDNA using the RevertAid First Strand cDNA Synthesis Kit (Thermo Fisher Scientific, Waltham, MA, USA). RT-PCR was performed using SYBR Green Master Mix (Roche Diagnostics, Basel, Switzerland) according to the manufacturer’s instructions. The primers used in this study were as follows: Cyr61 forward: TCCAGCCCAACTGTAAACATCA reverse: GGACACAGAGGAATGCAGCC; GAPDH forward: CACATGGCCTCCAAGGAGTA reverse: TGAGGGTCTCTCTCTTCCTCTTGT; Bcl-2 forward: CTGGTGGGAGCTTGCATCAC reverse: ACAGCCTGCAGCTTTGTTTC; Bcl-xl forward: TCAGGCTGCTTGGGATAAAGAT reverse: AGAGGCTTCTGGAGGACATTTG; Survivin forward: TGACGACCCCATAGAGGAACA reverse: CGCACTTTCTCCGCAGTTTC; XIAP forward: TTGAGGAGTGTCTGGTAAG reverse: CCATTCGTATAGCTTCTTGT.

### Apoptosis assay

Cell apoptosis was evaluated using a FACSCanto II cytometer (BD Biosciences, San Jose, CA) and the Annexin V‐APC Apoptosis Detection Kit (BD Biosciences, Franklin Lakes, NJ, USA) according to the manufacturers’ instructions. Cells (5.0 × 10^5^) cells were washed with ice‐cold PBS, re-suspended in binding buffer (195 μL), and stained with APC-conjugated anti‐annexin-V antibody (5 µL) for 10 min at 25 °C. Unbound annexin-V antibody was washed off using binding buffer. The percentage of apoptotic Nalm-6 cells (annexin-V positive) was determined using flow cytometry analysis.

### Cyr61 down-regulation

Cyr61 knockdown was performed as previously described (Song et al. 2019). Nalm-6 cells (5 × 10^4^ cells/mL) were infected by a lentivirus carrying shNC or shCyr61 (Shanghai GeneChem Co., Ltd, Shanghai, China). Upon ensuring > 95% cell infection efficiency, 4 μg/mL puromycin (Sigma-Aldrich, St. Louis, MO) was used for selection for 5 days. Cyr61 knockdown efficiency was measured using western blot analysis and the frequency of GFP^+^ cells was measured using flow cytometry.

### Cyr61 overexpression

Lentiviral particles containing the Cyr61 sequence were purchased from Shanghai GeneChem Co., Ltd. (Shanghai, China). Nalm-6 cells (5 × 10^4^ cells/mL) were infected with the Cyr61-containing lentiviral particles for 18–24 h according to the manufacturer’s protocol. Upon ensuring > 95% infection efficiency of Nalm-6 cells, 4 μg/mL puromycin (Sigma-Aldrich, St. Louis, MO) was used for selection for 5 days. Cyr61 knockdown efficiency was measured using western blot analysis and the frequency of GFP^+^ cells was measured using flow cytometry.

### Statistical analysis

SPSS 22.0 statistical software (Version 22.0 SPSS, Chicago, IL; USA) was used for statistical analyses. All data were expressed as mean ± standard error of the mean (SEM). Student’s test was used to compare two groups and *P* < 0.05 was considered statistically significant (*P < 0.05; **P < 0.01).

## Results

### Cyr61 decreased the chemosensitivity of B-ALL cells to DNR

**Fig. 1:**
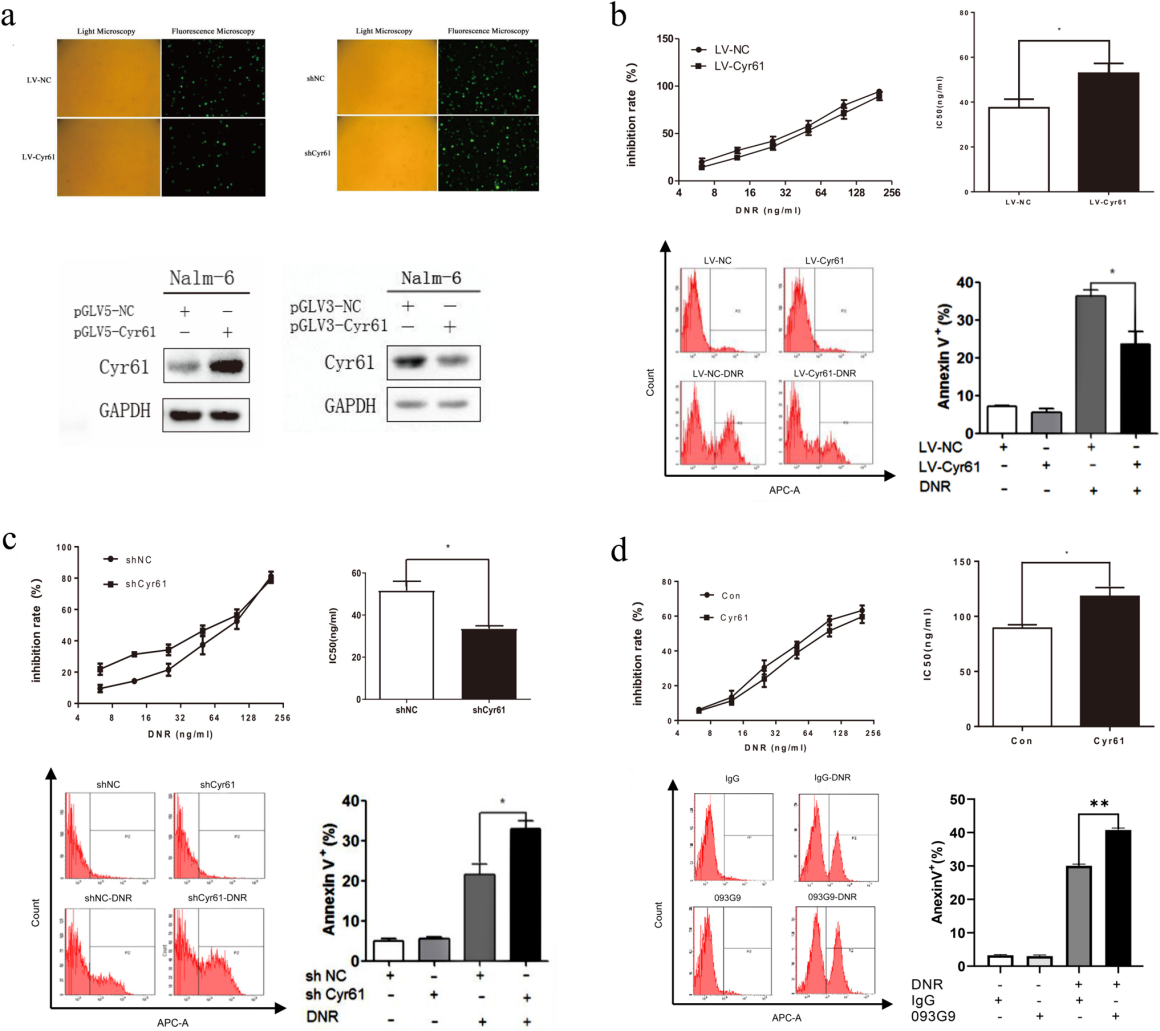
Cyr61 decreased the chemosensitivity of B cell acute lymphoblastic leukemia (B-ALL) cells to daunorubicin (DNR). **a** Nalm-6 cells were infected with lentivirus for 72 h. The infection efficiency was determined using a fluorescence microscope. The Cyr61 protein levels in Nalm-6-shNC, Nalm-6-shCyr6, Nalm-6-LV-Cyr61, and Nalm-6-LV-NC cells were detected using western blotting. **b** Nalm-6-LV-Cyr61 and Nalm-6-LV-NC cells were treated with DNR, and the cell viability analyzed using cell counting kit-8 (CCK8) assays and the apoptotic rates determined using flow cytometric analysis. **c** Nalm-6-shNC and Nalm-6-shCyr6 cells were treated with DNR, and the cell viability analyzed using CCK8 assays and the apoptotic rates determined using flow cytometric analysis. **d** Nalm-6 cells were treated with DNR, exogenous Cyr61 with/without Cyr61 monoclonal antibody (1 μg/mL), and the cell viability analyzed using CCK8 assays and the apoptotic rates determined using flow cytometric analysis

Cyr61 overexpression and knockdown were constructed in B-ALL cell lines using lentivirus vector transfection and the efficacy was confirmed (Fig. 1a). Cell viability was assessed using a CCK8 assay and the results showed that Cyr61 overexpression in Nalm-6 cells led to a higher IC50 value to DNR and a lower DNR-induced apoptosis rate than that in the Nalm-6-LV-NC cell group, suggesting that Cyr61 overexpression reduced the chemosensitivity of Nalm-6 cells to DNR (Fig. 1b). Conversely, Cyr61 knockdown in Nalm-6 cells resulted in a lower IC50 value to DNR and a higher DNR-induced apoptosis rate than that in the Nalm-6-shNC cell group, indicating that Cyr61 knockdown enhanced the chemosensitivity of Nalm-6 cells to DNR (Fig. 1c). To further identify the effect of Cyr61 on the chemosensitivity of Nalm-6 cells to DNR, exogenous purified Cyr61 protein was used, and the results showed that exogenous Cyr61 increased the IC50 of B-ALL cells to DNR and decreased the DNR-induced apoptosis rate, which could be blocked by the Cyr61 monoclonal antibody (093G9) (Fig. 1d). These findings demonstrate that Cyr61 decreases the chemosensitivity of B-ALL cells to DNR.

### Cyr61 upregulated BCL-2 levels in B-ALL cells

**Fig. 2:**
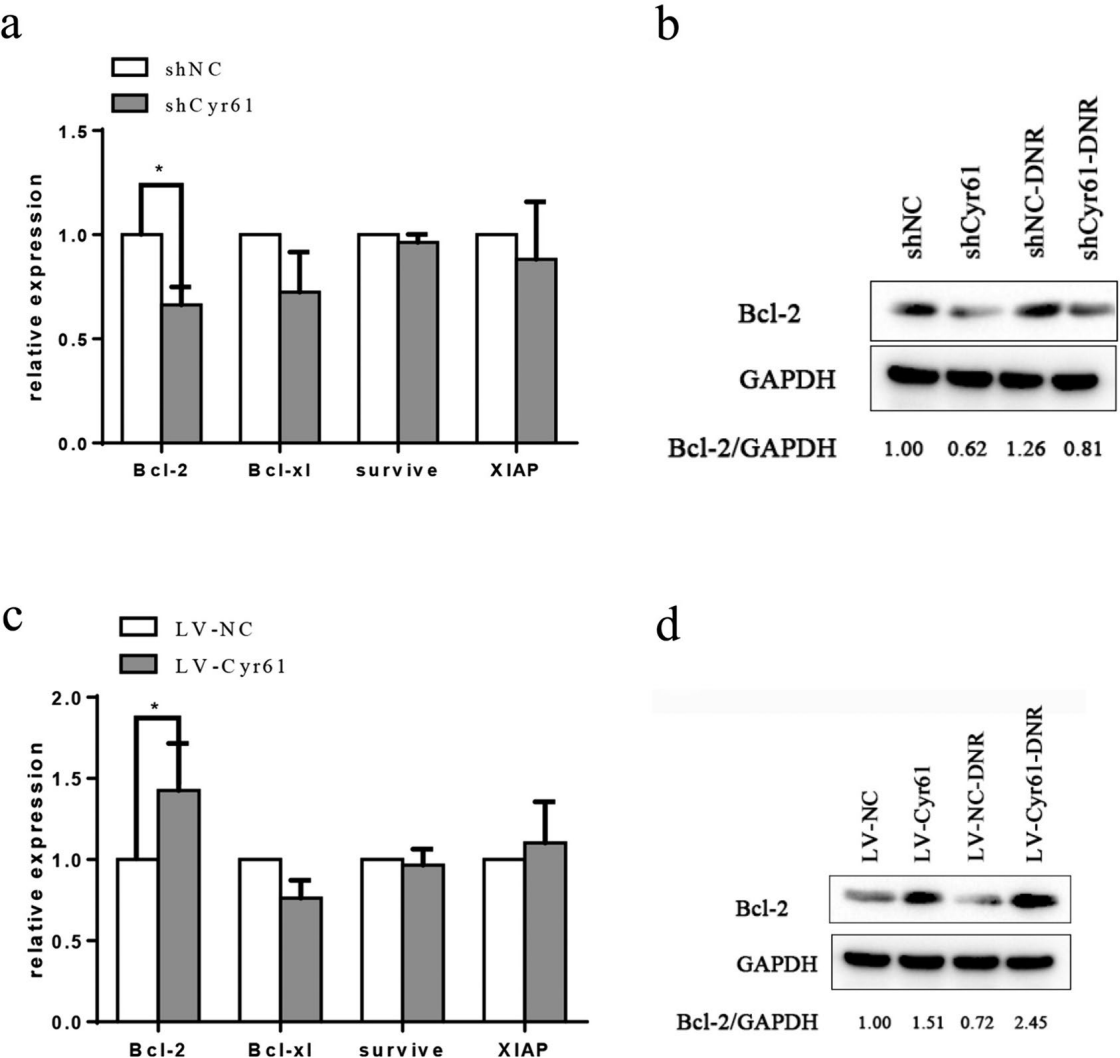
Cyr61 up-regulates B cell lymphoma-2 (BCL-2) levels in B cell acute lymphoblastic leukemia (B-ALL) cells. **a** Nalm-6-shCyr61 and Nalm-6-shNC cells were treated with daunorubicin (DNR) for 24 h, and Bcl-2, Bcl-xl, survivin, and X-linked inhibitor of apoptosis protein (XIAP) mRNA expression levels were detected using real‐time polymerase chain reaction (PCR). **b** Nalm-6-shNC and Nalm-6-shCyr61 cells were treated with DNR for 24 h, and BCL-2 protein production was detected using western blotting. **c** Nalm-6-LV-NC and Nalm-6-LV-Cyr61 cells were treated with DNR for 24 h, and the Bcl-2, Bcl-xl, survivin, and XIAP mRNA expression was detected using real‐time PCR. **d** Nalm-6-LV-NC and Nalm-6-LV-Cyr61 cells were treated with DNR for 24 h, and the BCL-2 protein production was detected using western blotting. The band intensity of BCL‐2 was quantified by densitometry and normalized to glyceraldehyde 3-phosphate dehydrogenase (GAPDH). Data represented the mean ± standard error of the mean (SEM) of at least three independent experiments. *P < 0.05, **P < 0.01

Cyr61 knockdown downregulated Bcl-2 expression, whereas Cyr61 overexpression upregulated Bcl-2 expression (Fig. 2a). However, Bcl-xl, Survivin, and XIPA expression remained significantly unchanged. In addition, western blot indicated that the Bcl-2 levels were significantly decreased in Cyr61-knockdown Nalm-6 cells (Fig. 2b) and increased in Cyr61-overexpression Nalm-6 cells (Fig. 2d). These results suggested that Cyr61 can upregulate Bcl-2 in B-ALL cells.

### DNR promoted Cyr61 production in B-ALL cells

**Fig. 3:**
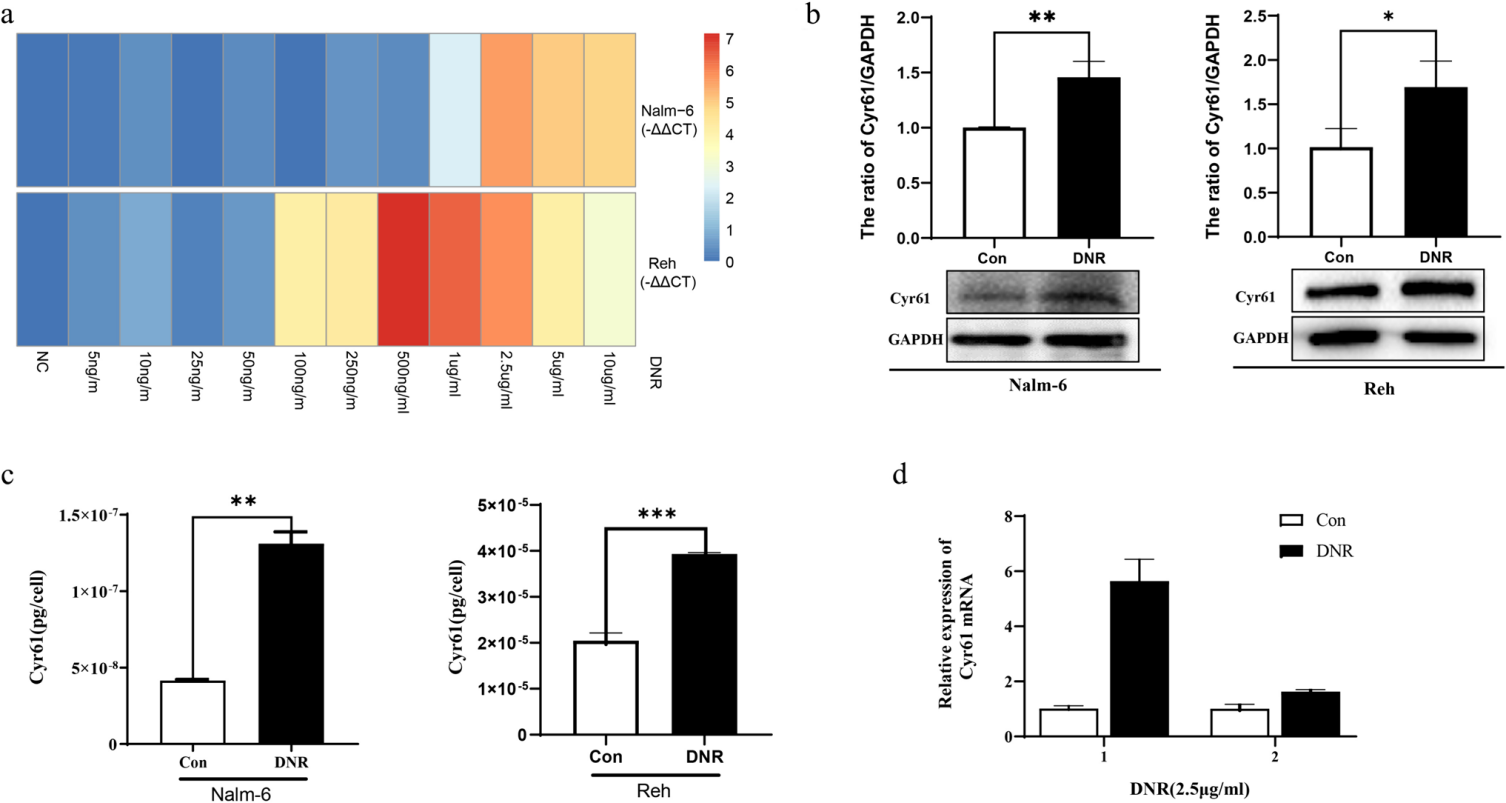
Daunorubicin (DNR) promoted Cyr61 production in B cell acute lymphoblastic leukemia (B-ALL) cells. **a** Nalm-6 and Reh cells were treated with different concentrations of DNR for 24 h, and Cyr61 mRNA expression was detected using real‐time polymerase chain reaction (PCR). **b** Nalm-6 and Reh cells were treated with 2.50 μg/mL DNR for 24 h, and Cyr61 protein levels detected using western blotting. The band intensity of Cyr61 was quantified by densitometry and normalized to glyceraldehyde 3-phosphate dehydrogenase (GAPDH). **c** Nalm-6 and Reh cells were treated with 2.50 μg/mL DNR for 24 h, and Cyr61 protein levels in cell culture supernatants were measured using enzyme-linked immunosorbent assay (ELISA). **d** Primary leukemic cells from two patients with B-ALL were isolated and treated with 2.50 μg/mL DNR for 24 h, and the Cyr61 mRNA expression detected using real‐time PCR. Data represented the mean ± standard error of the mean (SEM) of at least three independent experiments. *P < 0.05, **P < 0.01

We examined Cyr61 production in DNR-treated B-ALL cells using qRT-PCR and western blotting. Cyr61 mRNA and protein levels were significantly increased in DNR-treated Nalm-6 and Reh cells (Fig. 3a, b). To further validate this finding, we collected culture supernatants from DNR-treated B-ALL cells and examined the Cyr61 levels using ELISA. Cyr61 expressions were increased in the culture supernatants of DNR-treated B-ALL cells (Fig. 3c). Moreover, we collected primary B-ALL cells from patients and found that Cyr61 mRNA levels were significantly increased in DNR-treated primary B-ALL cells (Fig. 3d). Together, these results suggested that DNR promotes Cyr61 production in B-ALL cells.

### NF-κB signaling pathway involved in DNR-induced Cyr61 production in B-ALL cells

**Fig. 4:**
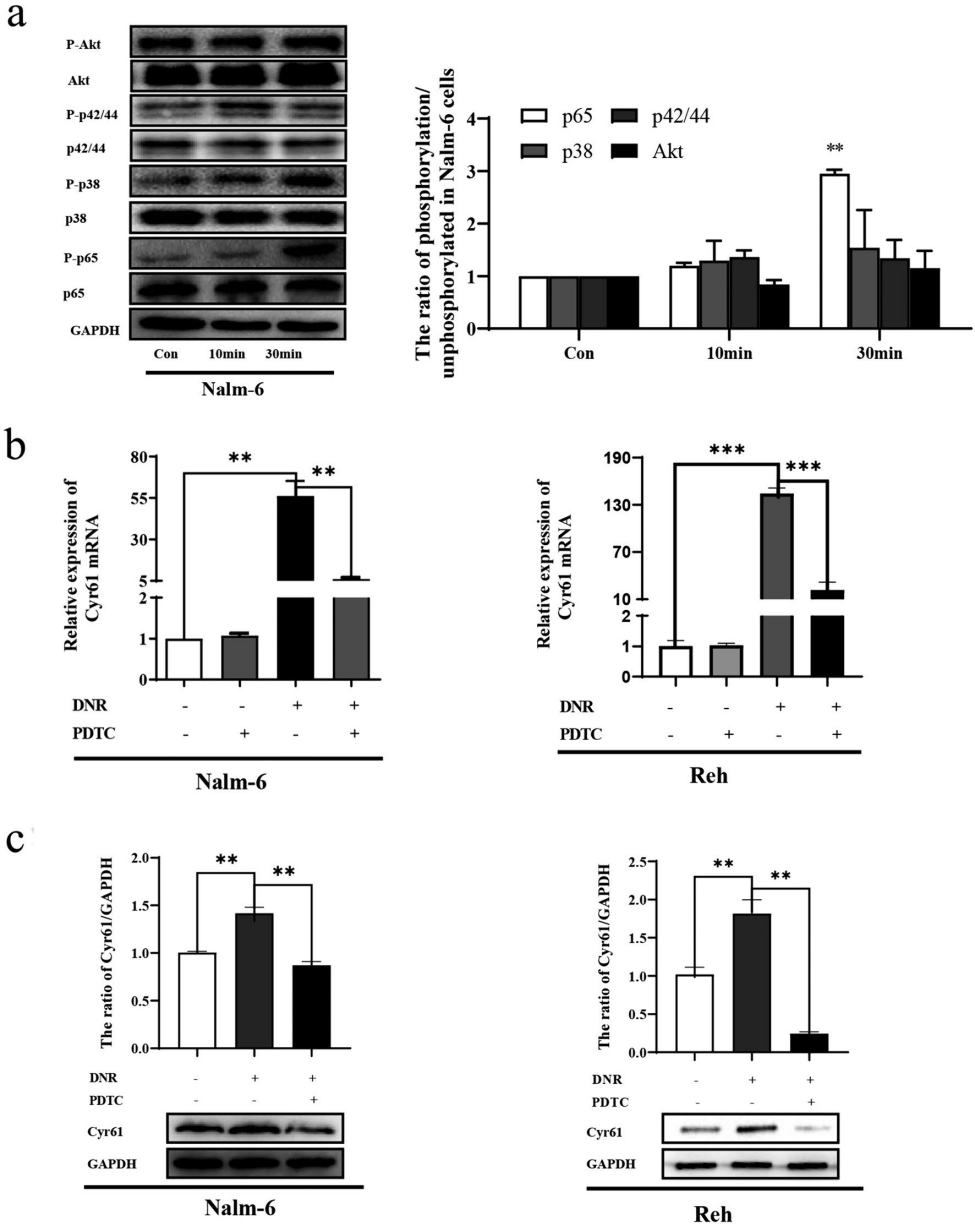
Nuclear factor kappa B (NF-κB) signaling pathway involved in daunorubicin (DNR)-induced Cyr61 production in B cell acute lymphoblastic leukemia (B-ALL) cells. **a** Nalm-6 cells were treated with DNR for 10 min and 30 min, and the PI3K/AKT, p38 MAPK, ERK (p44/p42), and NF-κB pathway phosphorylation detected using western blotting. **b** Nalm-6 and Reh cells were treated with DNR with or without 40μΜ pyrrolidine dithiocarbamate (PDTC) for 24 h. The Cyr61 mRNA levels were detected using real‐time polymerase chain reaction (PCR). **c** Nalm-6 and Reh cells were treated with DNR with or without 40 μΜ PDTC for 24 h, and the Cyr61 protein levels detected using western blotting. The band intensity of Cyr61 was quantified by densitometry and normalized to glyceraldehyde 3-phosphate dehydrogenase (GAPDH). Data represented the mean ± standard error of the mean (SEM) of at least three independent experiments. *P < 0.05, **P < 0.01

To address the mechanism by which DNR promotes Cyr61 production in B-ALL cells, we evaluated the phosphorylation of the AKT, p38, ERK1/2, and NF-κB pathways in DNR-treated B-ALL cells. The phosphorylation of NF-κB/p65 was markedly increased in the presence of DNR, while the phosphorylation of AKT, p38, and ERK1/2 was not significantly affected compared with that in the control group (Fig. 4a). Further analysis showed that pyrrolidine dithiocarbamate (PDTC) (a NF-κB activation inhibitor) decreased Cyr61 mRNA expression in DNR-treated Nalm-6 and Reh cells (Fig. 4b). Similar results were found using western blotting (Fig. 4c). Our results indicated that DNR can effectively upregulate Cyr61 production via NF-κB pathway activation.

### ATM signaling pathway promoted NF-κB activation in DNR-induced Cyr61 production in B-ALL cells

**Fig. 5:**
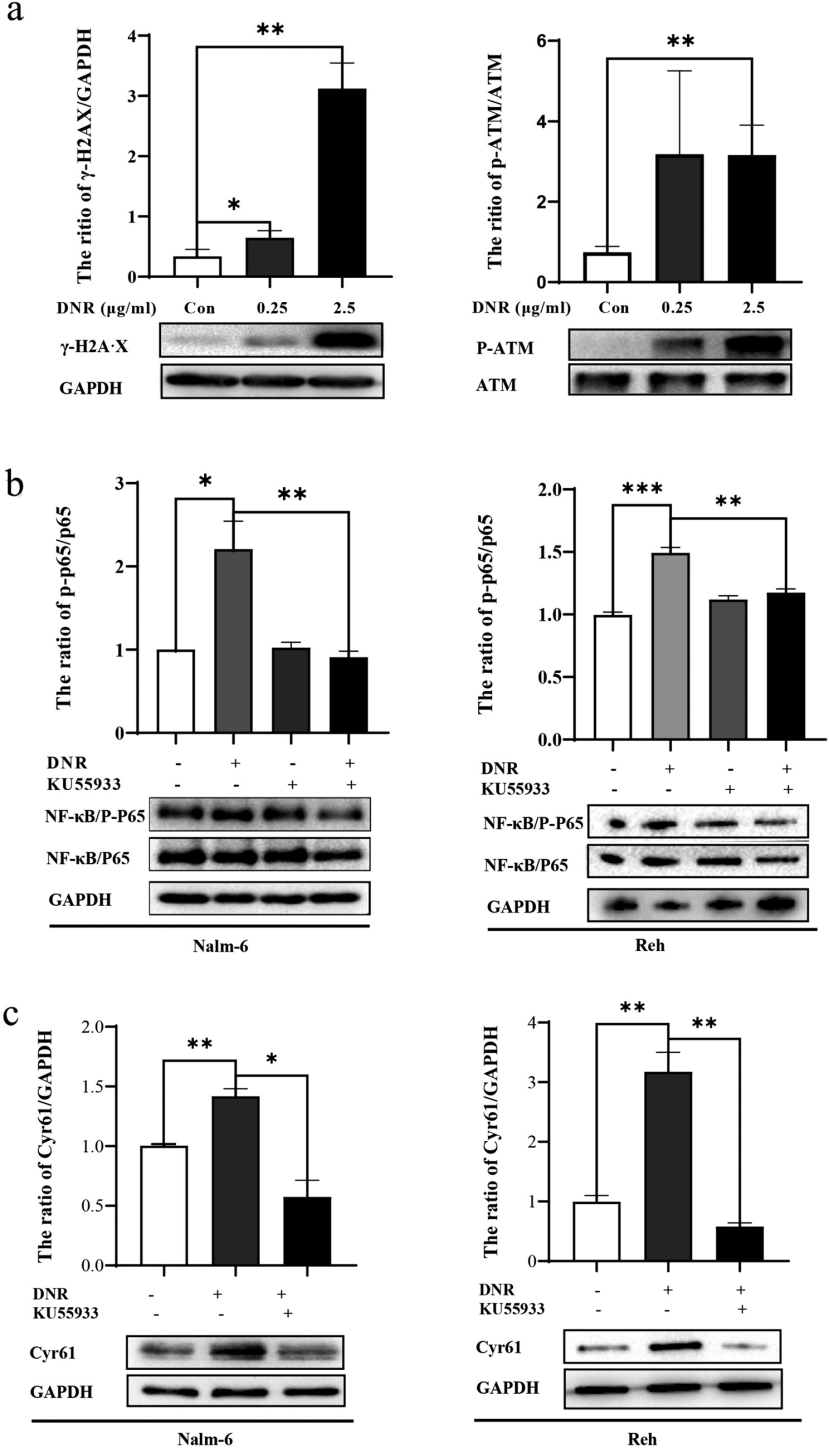
Ataxia telangiectasia mutated (ATM) signaling pathway promoted nuclear factor kappa B (NF-κB) activation in daunorubicin (DNR)-induced Cyr61 production in B cell acute lymphoblastic leukemia (B-ALL) cells. **a** Nalm-6 cells were treated with DNR for 30 min, and H2A·X (left panel) and ATM (right panel) phosphorylation was detected using western blotting. **b** Nalm-6 and Reh cells were treated with DNR with or without 10 μM KU55933 for 30 min, and the NF‐κB/p65 phosphorylation levels detected using western blotting. **c** Nalm-6 and Reh cells were treated with DNR with or without 10 μM KU55933 for 24 h, and the Cyr61 protein levels detected using western blotting. The band intensity of Cyr61 was quantified by densitometry and normalized to glyceraldehyde 3-phosphate dehydrogenase (GAPDH). AS p-ATM and ATM are very big. To detect them in western blotting is not that easy. It is convention to introduce the molecular-weight marker (MM) above and below the detected protein (exception of for ATM because too high for a conventional MM. Therefore, the MM below is enough). Data represented the mean ± standard error of the mean (SEM) of at least three independent experiments. *P < 0.05, **P < 0.01

DNA damage repair proteins were assessed using western blotting and the results showed that the phosphorylation of ATM and H2A·X was significantly increased in DNR-treated Nalm-6 cells (Fig. 5a). Next, we investigated whether the ATM/NF-κB signaling pathway is involved in Cyr61 expression regulation in B-ALL cells. KU55933 (a specific ATM phosphorylation inhibitor) decreased NF-κB phosphorylation in DNR-treated B-ALL cells (Nalm-6 and Reh cells) (Fig. 5b). Simultaneously, the Cyr61 production was markedly decreased in the presence of DNR together with KU55933 than that in DNR alone (Fig. 5c). Collectively, ATM signaling pathway promoted NF-κB activation in DNR-induced Cyr61 production in B-ALL cells.

## Discussion

Despite recent advances in B-ALL treatment, relapse and drug resistance remain major challenges (Jabbour et al. 2015). In this study, we reported that DNR effectively induced Cyr61 production in B-ALL cells via the ATM-dependent NF-κB pathway, and increased Cyr61 decreases the chemosensitivity of B-ALL cells to DNR via Bcl-2 upregulation. Our findings suggest that targeting Cyr61 or related ATM signaling pathways may present a promising treatment strategy to enhance the chemosensitivity of patients with B-ALL.

Cyr61 is abnormally expressed in tumors, and dysregulated Cyr61 promotes tumor proliferation and mediates drug resistance in tumor cells (Zhu et al. 2016; Xie et al. 2001, 2004; Lin et al. 2005; Gery et al. 2005). Our previous study confirmed that Cyr61 levels are elevated in the bone marrow of patients with ALL, and increased Cyr61 promotes ALL cell survival and decreases the chemosensitivity of ALL cells to cytarabine (Cao et al. 2019). Not surprisingly, in this study, we showed that Cyr61 decreases the chemosensitivity of B-ALL cells to DNR via Bcl-2 production. Our findings are consistent with previous results that the Bcl-2 pathway is involved in Cyr61-induced cytarabine resistance in ALL cells (Cao et al. 2019). According to the present and previous studies (Cao et al. 2019; Song et al. 2019), Cyr61 plays important roles in the drug resistance of B-ALL, and blocking the Cyr61 pathway may improve B-ALL treatment.

DNR is a common first-line chemotherapy drug used to treat ALL. In this study, we found that DNR can promote Cyr61 production in B-ALL cells. Chemotherapy can induce drug resistance by stimulating the release of various cytokines that shield tumor cells from the cytotoxic effects of chemotherapy agents (Chen et al. 2019; Duan et al. 2014; Anthony and Link 2014). Our finding suggests that Cyr61 upregulation in B-ALL cells in response to DNR may represent a self-protective mechanism for B-ALL cell survival. In other words, increased Cyr61 in response to DNR may protect B-ALL cells from its cytotoxic effects. Thus, DNR-induced Cyr61 contributes to B-ALL therapy resistance, which presents a possible reason for relapse and drug resistance in patients with B-ALL.

We next explored the molecular mechanism of Cyr61 production in B-ALL cells. The NF-κB signal pathway is involved in regulating Cyr61 expression in multiple cancer cell types (Lin et al. 2004; Lee et al. 2012). First, we found that NF-κB activation was markedly increased in DNR-treated B-ALL cells and NF-κB activation inhibitor can decrease Cyr61 production, suggesting that NF-κB signaling pathway is involved in DNR-induced Cyr61 production in B-ALL cells. Chemotherapy drug induces DNA damage and subsequent DNA damage repair (DDR) response. ATM, a key molecule in the DDR response, can promote anti-apoptotic gene expression via NF-κB activation in leukemic cells (Panta et al. 2004) (Bagci et al. 2006). We next explore ATM effects on DNR-induced Cyr61 in B-ALL cells using specific chemical inhibitor. As expected, blockade of ATM kinase activity markedly decreased the NF-κB activation and Cyr61 production. Our study findings are consistent with previous reports in which DNR/doxorubicin activated the NF-κB signaling pathway and subsequently conferred chemotherapy drug resistance to malignant cells in breast and ovarian cancer (Vasiyani et al. 2022; Lee et al. 2018; Fang et al. 2014; Esparza-López et al. 2013). Collectively, DNR induced Cyr61 production via ATM/NF-κB signaling in B-ALL cells.

However, our study is limited to cell-based in vitro experiments, and lacked in vivo experimental study. Considering the complexity of the bone marrow microenvironment with a variety of cells and cytokines, in vitro studies seem to be insufficient to elucidate the role of Cyr61 in the drug resistance of B-ALL. Thus, further in vivo studies should be carried out to confirm the role of Cyr61 in B-ALL cell resistance and the signaling pathways underlying DNR-induced Cyr61 production in B-ALL cells.

In summary, our results for the first time provided compelling evidence that DNR promoted Cyr61 production via an ATM-dependent NF-κB pathway in B-ALL cells**.** Moreover, increased Cyr61 could protect B-ALL cells against chemo-induced apoptosis, which led to increased drug resistance of B-ALL cells. Our findings suggest that targeting Cyr61 or related ATM signaling pathways may present a promising treatment strategy to enhance the chemosensitivity of patients with B-ALL.

## Data Availability

All data generated or analyzed during this study are included in this article.

## Abbreviations

ALL: Acute lymphoblastic leukemia
B-ALL: B-acute lymphoblastic leukemia
Cyr61: Cysteine‐rich protein 61
DNR: Daunorubicin
VCR: Vincristine
DFS: Disease-free survival
qRT-PCR: Quantitative real-time PCR
ELISA: Enzyme-linked immunosorbent assay
IL-6: Interleukin-6
DMSO: Dimethyl sulfoxide
XIAP: X-linked inhibitor of apoptosis
NF‐κB: Nuclear factor kappa B
PI3K/AKT: Phosphatidylinositol-3-kinase
ERK: Extracellular signal-regulated kinase
SEM: Standard errors of the mean
CCK-8: Cell Counting Kit-8
OD: Optical density
IC50: 50% Inhibitory concentration
ATM: Ataxia telangiectasia mutated
DDR: DNA-damage respond
